# The Long Non-coding RNA TMPO-AS1 Promotes Bladder Cancer Growth and Progression *via* OTUB1-Induced E2F1 Deubiquitination

**DOI:** 10.3389/fonc.2021.643163

**Published:** 2021-03-18

**Authors:** Yeyu Zhang, Yuxing Zhu, Mengqing Xiao, Yaxin Cheng, Dong He, Jianye Liu, Liang Xiang, Lian Gong, Zhanwang Wang, Liping Deng, Ke Cao

**Affiliations:** ^1^Department of Oncology, The Third Xiangya Hospital of Central South University, Changsha, China; ^2^Department of Respiratory, The Second People's Hospital of Hunan Province, Changsha, China; ^3^Department of Urology, The Third Xiangya Hospital of Central South University, Changsha, China

**Keywords:** long non-coding RNA, bladder cancer, TMPO-AS1, E2F1, OTUB1

## Abstract

**Background:** Increasing evidence indicates that long non-coding RNAs (lncRNAs) play crucial roles in cancer tumorigenesis and progression. TMPO antisense RNA 1 (TMPO-AS1) has been found to be involved in several cancers by acting as a competing endogenous RNA. However, the potential roles of TMPO-AS1 in bladder cancer (BC) and the potential interactions with proteins remain poorly understood.

**Methods:** The expression of the lncRNA TMPO-AS1 was evaluated *via* bioinformatic analysis and further validated by quantitative real-time PCR (qRT-PCR). Loss- and gain-of-function assays were performed to determine the biological functions of TMPO-AS1 in BC cell proliferation, migration, and invasion. Moreover, chromatin immunoprecipitation, Western blotting, and fluorescence *in situ* hybridization, as well as RNA pull-down, RNA immunoprecipitation, and luciferase reporter assays, were conducted to explore the upstream and downstream molecules interacting with TMPO-AS1.

**Results:** TMPO-AS1 is upregulated in BC. Functional experiments demonstrated that TMPO-AS1 promotes cell proliferation, migration, and invasion in BC and inhibits cell apoptosis *in vivo* and *in vitro*. Mechanically, E2F1 is responsible for TMPO-AS1 upregulation. Additionally, TMPO-AS1 facilitates the interaction of E2F1 with OTU domain-containing ubiquitin aldehyde binding 1 (OTUB1), leading to E2F1 deubiquitination and stabilization; therefore, TMPO-AS1 promotes BC malignant phenotypes. Furthermore, rescue experiments showed that TMPO-AS1 promotes BC growth in an E2F1-dependent manner.

**Conclusions:** Our study is the first to uncover the novel TMPO-AS1/E2F1 positive regulatory loop important for the promotion of BC malignant behaviors. The TMPO-AS1/E2F1 loop should be considered in the quest for new BC therapeutic options.

## Introduction

Bladder cancer (BC) is the most common malignant tumor of the urinary system worldwide (1); about 549,393 new cases and 199,922 cancer-related deaths were reported in 2018 (2). The majority of BC cases are urothelial cell carcinomas. Of note, urothelial BC can be categorized into non-muscle-invasive BC (NMIBC) and muscle-invasive BC (MIBC). Approximately 75% of patients with BC exhibit NMIBC with high recurrence and progression, while the remaining 25% of patients with BC present with MIBC and have a poor prognosis (3). Although therapies, including transurethral resection, cystectomy, chemotherapy, radiation, and immunotherapy, have contributed to the reduction of BC-associated morbidity/mortality, the 5-years survival rate of patients with BC have hardly improved (4). Therefore, it is urgent to explore the molecular mechanisms and biomarkers of BC to develop better diagnostic, monitoring, and therapeutic approaches and reduce the disease burden.

Long non-coding RNAs (lncRNAs) are a class of non-coding RNAs longer than 200 nucleotides with limited protein-coding potential (5). LncRNAs are known to influence several biological and pathological processes, such as cell proliferation, metastasis, drug resistance, and metabolism and are involved in multiple diseases, particularly in cancer (6–8). Previous studies have suggested that numerous lncRNAs are involved in BC (9). Recent studies have demonstrated that TMPO antisense RNA 1 (TMPO-AS1) serves as a competing endogenous RNA to sponge microRNAs (miRNAs) in multiple carcinomas, including hepatocellular carcinoma, thyroid cancer, lung adenocarcinoma, and breast cancer (10–13). However, whether TMPO-AS1 interacts with other molecules and plays a role in BC is still unknown.

Besides regulating the transcription of mRNAs, transcription factors (TFs) are also involved in the regulation of the transcription of lncRNAs (14). E2F1, a member of the E2F transcription factor family consisting of eight proteins, is a transcription activator essential for the regulation of cell cycle, apoptosis, cell proliferation, and DNA damage response (15). Studies have demonstrated that E2F1 can modulate the expression of lncRNAs (16). However, little is known on the E2F1-mediated regulation of lncRNAs in BC. Of note, the stabilization of TFs can be modulated by ubiquitination and deubiquitination (17). However, no lncRNAs have been linked to E2F1 deubiquitination.

Here, we focus on the roles of TMPO-AS1 in BC carcinogenesis and investigate the protein upstream and downstream of TMPO-AS1. We demonstrate that E2F1 activates the transcription of TMPO-AS1, which, in turn, facilitates the interaction of E2F1 with OTU domain-containing ubiquitin aldehyde binding 1 (OTUB1), a deubiquitinase; consequently, the E2F1 protein levels are increased *via* stabilization, promoting BC malignant phenotypes. Therefore, the TMPO-AS1/E2F1 positive feedback loop should be considered as a novel target for the treatment of BC.

## Materials and Methods

### Bioinformatic Analysis

The expression data on lncRNA TMPO-AS1 in 33 types of human cancers were obtained from the Gene Expression Display Server GEDS (http://bioinfo.life.hust.edu.cn/web/GEDS/) (18). The expression of TMPO-AS1 in BC tissues and in normal tissues was analyzed using The Cancer Genome Atlas (TCGA) BLCA RNA-seq data retrieved from the UCSC XENA (https://xena.ucsc.edu), TANRIC (https://ibl.mdanderson.org/tanric/_design/basic/analysis.html) (19), and Gene Expression Omnibus (GEO, www.ncbi.nlm.nih.gov, GSE133624 and GSE120736 datasets) databases. The prognostic value of TMPO-AS1 was evaluated using GEPIA 2 (http://gepia2.cancer-pku.cn/#index). Additionally, hTFtarget (http://bioinfo.life.hust.edu.cn/hTFtarget#!/) and ChIPBase v2.0 (http://rna.sysu.edu.cn/chipbase/index.php) were used to find out the potential TFs of TMPO-AS1 (20, 21). The JASPAR (http://jaspar.genereg.net) 2018 database was used to identify the E2F1-TMPO-AS1 binding profile (22). Genes co-expressed with TMPO-AS1 (TCGA-BLCA dataset) were defined as those with the correlation coefficients ≥0.6 and *p-*values <0.01 using Co-LncRNA (http://bio-bigdata.hrbmu.edu.cn/Co-LncRNA/). (23). A pathway enrichment analysis was conducted using Metascape (https://metascape.org/gp/index.html#/main/step1) (24). The interactions between E2F1, OTUB1, and TMPO-AS1 were predicted *via* Agostini et al. introduced catRAPID (http://s.tartaglialab.com/page/catrapid_group), a server to identify the interaction of RNA and protein (25) and Tuvshinjargal et al. developed a web server named PRIdictor (http://bclab.inha.ac.kr/pridictor) to reveal mutual binding in protein and RNA (26). Additionally, the protein interactions between E2F1 and OTUB1 were predicted using HDOCK (http://hdock.phys.hust.edu.cn) (27). Last but not least, the ubiquitination sites of E2F1 were predicted using UbPred (http://www.ubpred.org) (28).

### Clinical Samples

Resected BC and normal adjacent specimens were collected from patients with BC admitted to the Third Xiangya Hospital, Central South University, Hunan, China, from 2016 to 2018; all patients provided written informed consent. Six pairs of BC and paired adjacent normal tissues were stored in liquid nitrogen at −80°C. This study was approved by the ethics committee of the Third Xiangya Hospital, Central South University, Hunan, China.

### Cell Culture and Treatments

The human BC cell lines, namely 5637, T24, and RT4, were obtained from American Type Culture Collection (ATCC; Rockville, MD, USA). BIU87 and EJ were purchased from the Advanced Research Center of Central South University (Changsha, China). Cells were cultured in Dulbecco's modified Eagle's medium (DMEM; Invitrogen, Carlsbad, CA, USA) containing 10% fetal bovine serum (FBS; Gibco, Thermo Fisher Scientific, Waltham, MA, USA), 1 mmol/L glutamine, and 100 U/ml penicillin at 37°C in an incubator with 5% CO_2_. The protein synthesis inhibitor cycloheximide (10 μg/ml, C1998; Millipore, Sigma-Aldrich, St Louis, MO, USA) and proteasome inhibitor MG132 (20 μM, S2619; Selleck, Houston, TX, USA) were used to examine the ubiquitin proteasome-related protein degradation.

### Quantitative Real-Time PCR

Total RNA was extracted from BC tumor tissues, the paired adjacent normal tissues, and BC cells (T24 and RT4) using TRIzol (Invitrogen, Carlsbad, CA, USA) according to the instructions in the PrimeScript RT Reagent Kit (TaKaRa, Dalian, China). The quantitative real-time PCR (qRT-PCR) was performed using the SYBR Green PCR Master Mix (Toyobo, Osaka, Japan) as per the instructions of the manufacturer. The relative expression of genes was determined using the 2^−ΔΔCT^ method, and the expression was normalized to that of β-actin. All experiments were performed in triplicate. The primer sequences used in this study are listed in Table 1.

**Table 1:** Primers used for qRT-PCR, siRNAs oligonucleotides, shRNA oligonucleotides and ChIP.

**Primers used for qRT-PCR**	
TMPO-AS1-F	AGAGCCGAACTACGAACCAA
TMPO-AS1-R	CTGTCCCTTATCGGCGTCT
E2F1-F	ACGTGACGTGTCAGGACCT
E2F1-R	GATCGGGCCTTGTTTGCTCTT
β-actin-F	CATGTACGTTGCTATCCAGGC
β-actin-R	CTCCTTAATGTCACGCACGAT
U1-F	GGGAGATACCATGATCACGAAGGT
U1-R	CCACAAATTATGCAGTCGAGTTTCCC
**siRNAs oligonucleotides**	
TMPO-AS1-F	GAGCCGAACUACGAACCAACU
TMPO-AS1-R	UUGGUUCGUAGUUCGGCUCUG
E2F1-F	ACCUCUUCGACUGUGACUUUG
OTUB1-F	AGCGACUCCGAAGGUGUUAAC
OTUB1-R	GUUAACACCUUCGGAGUCGCU
Negative control-F	UUCUCCGAACGUGUCACGUTT
Negative control-R	ACGUGACACGUUCGGAGAATT
**shRNA oligonucleotides**	
TMPO-AS1-F	GAGCCGAACTACGAACCAACT
TMPO-AS1-R	TTGGTTCGTAGTTCGGCTCTG
**ChIP**	
TMPO-AS1-F	CAACAAGTGCGACACTCCAT
TMPO-AS1-R	GTGTGGAGGGCTTTTTGAAC
GAPDH-F	TACTAGCGGTTTTACGGGCG
GAPDH-R	TCGAACAGGAGGAGCAGAGAGCGA

*BC, bladder cancer; lncRNAs, long non-coding RNAs; TMPO-AS1, TMPO antisense RNA 1; E2F1, E2F transcription factor 1; OTUB1, OTU domain-containing ubiquitin aldehyde binding 1; NMIBC, non-muscle-invasive bladder cancer; MIBC, muscle-invasive bladder cancer; miRNA, microRNA; TF, transcription factor; TCGA, The Cancer Genome Atlas; ChIP, chromatin immunoprecipitation; FISH, fluorescence in situ hybridization; IHC, immunohistochemistry; qRT-PCR, quantitative real-time PCR; RIP, RNA immunoprecipitation; Co-IP, co-immunoprecipitation*.

### Cell Transfection

For *in vitro* functional assays, TMPO-AS1, E2F1, and OTUB1 overexpression plasmids and small interfering RNAs, as well as the empty vectors, were designed by GeneChem Co., Ltd (Shanghai, China) and transfected into T24 and RT4 cells using the Lipofectamine 3000 Reagent (Invitrogen, Carlsbad, CA, USA). For *in vivo* xenograft experiments, RT4 cells were stably transfected with empty lentiviral vectors, sh-TMPO-AS1 (designed according to the sequence of si-TMPO-AS1) or sh-TMPO-AS1, together with E2F1 overexpressing lentiviral vectors purchased from GeneChem Co., Ltd. (Shanghai, China) according to the protocol of the manufacturer. The empty vectors were used as the negative control. The transfection efficiency was determined *via* qRT-PCR.

### Methyl Thiazolyl Tetrazolium Assay

The methyl thiazolyl tetrazolium (MTT) assay was conducted according to the instructions of the manufacturer. Briefly, the BC cells were plated into 96-well plates at a density of 1 × 10^4^ cells/well and incubated for 24 h. Later, 10 μl of the MTT Solution (Sigma Chemicals, St. Louis, MO, USA) was added to each well, and the cells were cultured at 37°C for 4 h. Furthermore, cell viability/proliferation was estimated *via* the measurement of the absorbance at 570 nm with the Epoch Microplate Spectrophotometer (BioTek Instruments Inc., Winooski, VT, USA).

### Colony Formation Assay

The colony formation assay was performed as previously described in a study of Zeng et al. (29). Briefly, T24 and RT4 cells, treated as described in the abovementioned study, were seeded into 6-well plates at a density of 1,000 cells/well and cultured for 2–3 weeks. Then, the cells were washed with FBS, fixed with 4% paraformaldehyde, stained with 1% crystal violet, and counted. Only colonies with more than 50 cells were considered.

### Cell Apoptosis Analysis

The cell apoptosis in BC cells (T24 and RT4 cells) was investigated *via* flow cytometry using the Annexin V-PE/7-AAD Kit (KA3809; Abnova, Wuhan, China) according to the protocol of the manufacturer.

### Wound Healing Assay

The wound healing assay was conducted as previously described (30). Briefly, cells were seeded into 6-well plates at a density of 1 × 10^5^ cells/well. Then, a sterile 200 μl pipette tip was used to scratch a straight line in the cell monolayer. Later, the cells were washed with FBS and cultured for 48 h. The scratch width was measured 48 h later.

### Transwell Assay

The transwell assay was conducted to evaluate cell migration and invasion. Briefly, 5 × 10^4^ BC cells (T24 and RT4) were suspended in the serum-free medium and seeded into the upper chamber of the Transwell Plates (8 μm pore; Corning, Corning, NY, USA) with Matrigel (BD Biosciences, San Jose, CA, USA), while the complete medium with 10% FBS was added to the lower chamber. After a 48-h incubation, the migrating cells were fixed with 4% paraformaldehyde, stained with 0.1% crystal violet (Sigma-Aldrich, St. Louis, MO, USA), and photographed under a microscope (DMB5-2231P1, DMB HK Ltd., Hong Kong, China).

### Western Blotting

Total proteins were extracted using the radioimmunoprecipitation assay (RIPA) buffer (Beyotime Biotechnology Inc., Shanghai, China) with the Protease Inhibitor Cocktail (Roche, Basel, Switzerland). The protein concentration was measured using a bicinchoninic acid (BCA) kit (Thermo Fisher Scientific, Waltham, MA, USA). The protein samples were resolved *via* sodium dodecyl sulfate polyacrylamide gel electrophoresis (SDS-PAGE) and transferred onto polyvinylidene fluoride (PVDF) membranes. Later, the membranes were blocked in *phosphate-buffered saline* (PBS) containing 5% skim milk powder at room temperature for 1 h and incubated with the primary antibodies at 4°C overnight, followed by the secondary antibodies. The protein bands were visualized using the Pierce^®^ ECL Western Blotting Substrate Kit (32106; Thermo Fisher Scientific, Waltham, MA, USA) and normalized to the levels of β-actin as reference. The antibodies used in this study are listed in Supplementary Table 1.

### Luciferase Reporter Assay

Wild-type or mutant sequences of the E2F1 binding sites for the promoter of TMPO-AS1 were synthesized and inserted into the pGL3 vector (Promega, Madison, WI, USA). T24 and RT4 cells were seeded into 48-well plates and cotransfected with the above vectors along with E2F1 expression or control plasmids. About 48 h later, the luciferase activity was measured and analyzed using the Luciferase Reporter Assay System (Promega, Madison, WI, USA).

### Chromatin Immunoprecipitation Assay

The chromatin immunoprecipitation (ChIP) was performed using the EZ Magna ChIP™ Kit (Millipore, Burlington, MA, USA) according to the instructions of the manufacturer. Briefly, 1 × 10^7^ cells (T24 and RT4) were fixed with 1% formaldehyde and treated with 10% glycine. Later, the cross-linked chromatin was broken into small DNA fragments *via* sonication. The sonicated DNA was immunoprecipitated using antibodies against E2F1 or control rabbit immunoglobulin G (IgG) (Bioss Antibodies Inc., Woburn, MA, USA). qRT-PCR was performed to quantify the precipitated chromatin using the specific primers listed in Table 1.

### RNA Pull-Down Assay

TMPO-AS1 was transcribed *in vitro* and labeled *via* 3′-end biotinylation. The RNA pull-down assay was performed using the Pierce™ Magnetic RNA-Protein Pull-Down Kit (Thermo Fisher Scientific, Waltham, MA, USA). Briefly, the lysates of control or TMPO-AS1 overexpressing T24 and RT4 cells were incubated with control or biotinylated TMPO-AS1 at room temperature for 4 h, followed by the addition of streptavidin magnetic beads (Thermo Fisher Scientific, Waltham, MA, USA) at 4°C for 60 min with rotation. After three washing steps with washing buffer, the RNA-binding proteins were eluted using 50 μl elution buffer and analyzed *via* Western blotting.

### RNA Immunoprecipitation

RNA immunoprecipitation was performed using the EZ-Magna RIP™ RNA-Binding Protein Immunoprecipitation Kit (Millipore, Burlington, MA, USA) based on the instructions of the manufacturer. Briefly, cell extracts were incubated with magnetic beads conjugated with antibodies against SNRNP70 (Cat.# CS203216) and anti-E2F1 (Cat.# OM250777) or with normal rabbit IgG (Cat.# PP64B). Anti-SNRNP70 and normal rabbit IgG antibodies were used as positive and negative controls, respectively. The relative abundance of TMPO-AS1 was normalized to the amount of enriched U1snRNA *via* qRT-PCR.

### Co-immunoprecipitation

Cell lysates were incubated with primary antibodies against E2F1 (1:80, OM250777; Omnimabs, Alhambra, CA, USA) at 4°C overnight. Rabbit IgG antibodies (1:150, Bioss Antibodies Inc., Woburn, MA, USA) were used as the negative control. Later, the cell lysates were mixed with the protein A/G agarose (Cat.# P1012, Beyotime Biotechnology Inc., Jiangsu, China) at 4°C for 2 h, followed by centrifugation and washing steps. The precipitated complex was separated using the SDS-PAGE and analyzed *via* a Western blotting.

### Fluorescence *in situ* Hybridization

Fluorescence *in situ* hybridization (FISH) was performed using the Ribo™ Fluorescent *in situ* Hybridization Kit (RiboBio Co. Ltd., Guangzhou, China) following the instructions of the manufacturer. The TMPO-AS1 and 18S probes were synthesized and labeled with the Cy3 fluorescent dye. Fluorescence was detected under a Confocal Laser Microscope (SP5; Leica Microsystems, Wetzlar, Germany).

### Immunohistochemistry

The tissue sections obtained from paraffin-embedded tissues were dewaxed in xylene and rehydrated in an ethanol gradient. Later, tissues were incubated in 1% hydrogen peroxide and boiled in citrate buffer (10 mM, pH = 6.0) for 15 min. Subsequently, tissues were incubated with the primary antibodies against Ki-67 (1:1,000, 27309-1-AP; Proteintech, Chicago, IL, USA), E2F1 (1:200, OM250777; Omnimabs, Alhambra, CA, USA), and caspase-3 (1:200, 19677-1-AP; Proteintech, Chicago, IL, USA) at 4°C overnight, followed by incubation with horseradish peroxidase (HRP)-conjugated goat anti-rabbit secondary antibody (SP-9000, zsbio, Beijing, China) at room temperature for 30 min. Diaminobenzidine was used as chromogen; hematoxylin was used as the nuclear counterstain.

### Xenograft Mouse Model

All animal experiments were approved by the Animal Care and Use Committee of the Central South University. The 1 × 10^6^ RT4 cells transfected with empty vector, sh-TMPO-AS1, or sh-TMPO-AS1 together with E2F1-expressing lentiviral vectors were injected subcutaneously into the flanks of 4- to 6-week-old male BALB/c nude mice (*n* = 4 per group) obtained from the Shanghai Experimental Laboratory Animal Center (Shanghai, China). Tumor volumes were measured every 3 days and calculated as follows: tumor volume = (*D* × *d*^2^)/2, where *D* and *d* refer to the longest and shorter diameters, respectively. Mice were euthanized after 25 days.

### Statistical Analysis

All statistical analyses were performed using the GraphPad Prism Software, Version 8 (GraphPad Software, San Diego, CA, USA). Data are presented as the mean ± SD of at least three independent experiments. The relationship between E2F1 and TMPO-AS1 was analyzed using the Pearson's correlation coefficient. Significant differences were analyzed using the Student's *t*-test or the one-way ANOVA. Values of *p* < 0.05 were considered statistically significant.

## Results

### TMPO-AS1 Is Upregulated in Bc Tissues

**Figure 1:**
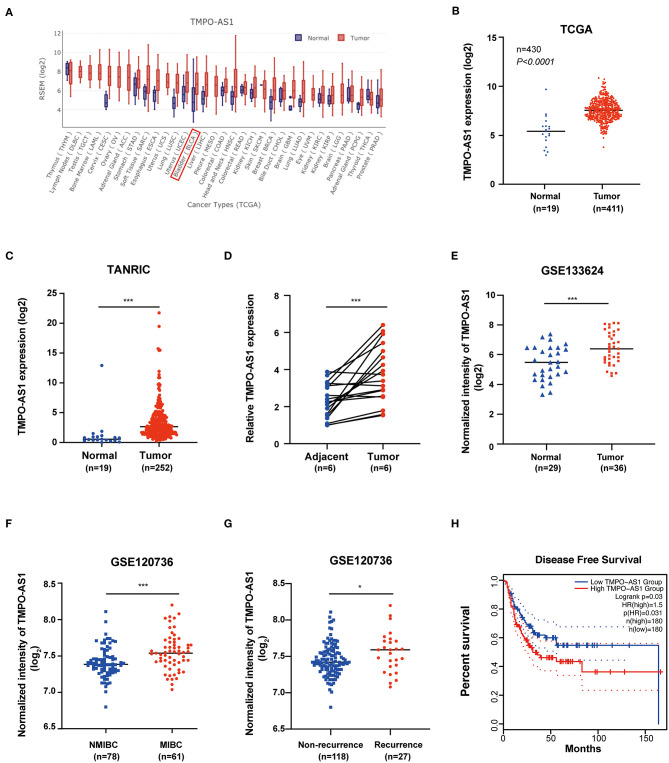
TMPO antisense RNA 1 (TMPO-AS1) is upregulated in bladder cancer (BC) tissues. **(A)** TMPO-AS1 is highly expressed in the majority of cancers according to the Gene Expression Display Server (GEDS). **(B,C)** The expression of TMPO-AS1 in BC vs. normal tissues was verified in data retrieved from TCGA and the TANRIC databases. **(D)** Quantitative real-time PCR (qRT-PCR) was conducted to validate the expression of TMPO-AS1 in six pairs of BC tissues and adjacent normal tissues collected in this study (replicates = 3). **(E–G)** The expression profile of TMPO-AS1 in BC as per two datasets retrieved for the GEO database: GSE133624 and GSE120736. **(H)** The disease-free survival curve shows that high levels of TMPO-AS1 expression are associated with poor prognosis of patients with BC (GEPIA). ^*^p < 0.05, ^***^p < 0.001.

To evaluate the expression of TMPO-AS1 in tumor and normal tissues, we used the online database GEDS; interestingly, we found that TMPO-AS1 is upregulated in multiple tumor tissues vs. normal tissues (Figure 1A). Consistently, the expression of TMPO-AS1 was upregulated in BC tissues compared with the normal tissues according to TCGA (Figure 1B) and TANRIC databases (Figure 1C). We further confirmed that the expression of TMPO-AS1 was higher in six BC tissues compared with that in the corresponding normal tissues *via* qRT-PCR (Figure 1D). Moreover, the integrative analysis of GSE133624 and GSE120736 showed that TMPO-AS1 was not only highly expressed in BC tissues (Figure 1E) but also exhibited higher levels in MIBC vs. NMIBC samples (Figure 1F). Of note, the higher expression of TMPO-AS1 was associated with the recurrence of BC (Figure 1G) and the advanced tumor stage (Supplementary Figure 1A). Furthermore, patients with BC of higher TMPO-AS1 expression levels were associated with shorter disease-free survival times (Figure 1H). Taken together, these data suggest that TMPO-AS1 is highly expressed in BC tissues, and it may serve as a potential prognostic biomarker in patients with BC.

### TMPO-AS1 Promotes the Proliferation, Migration, Invasion, and Survival of BC Cells *in vitro*

**Figure 2:**
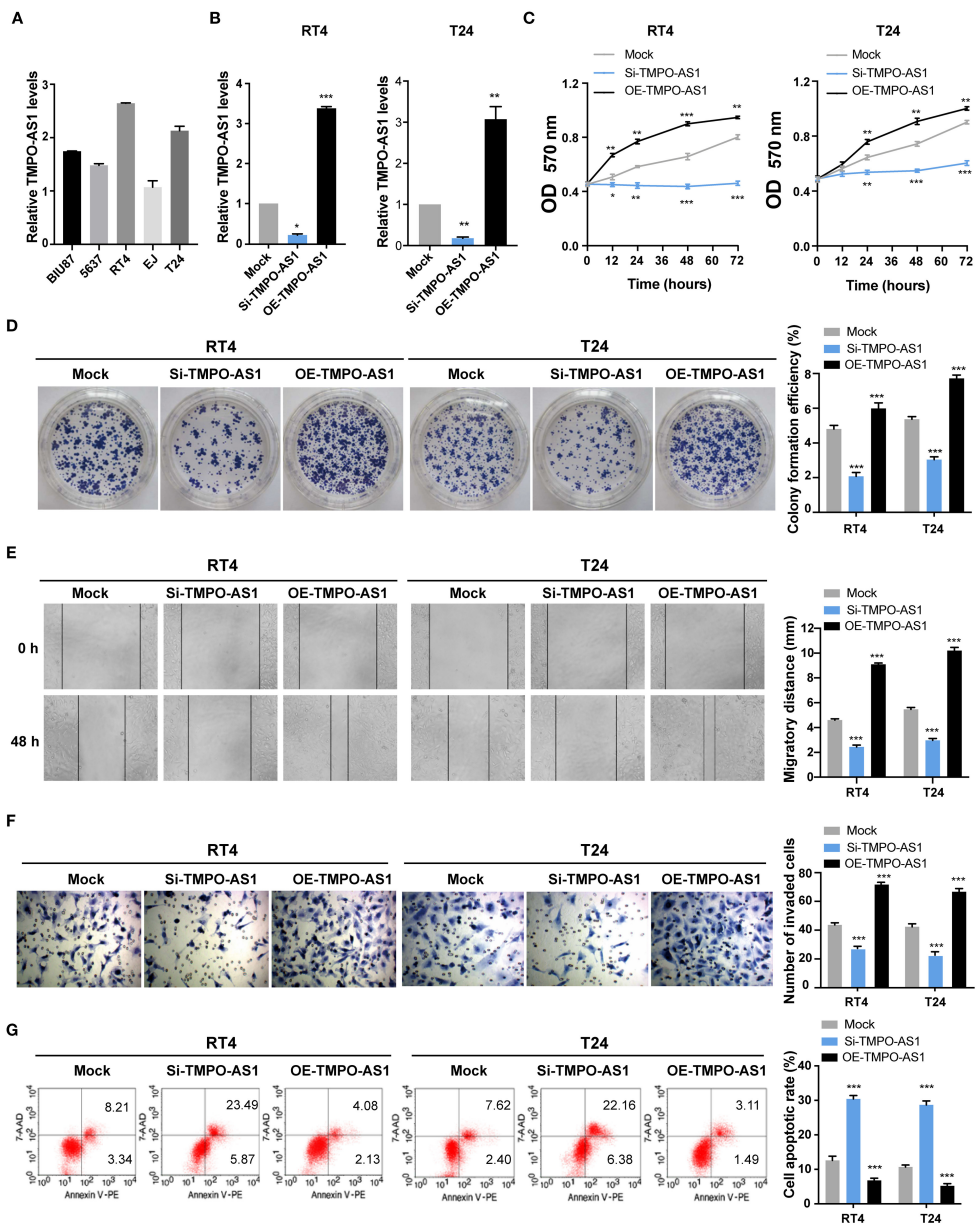
TMPO-AS1 promotes the proliferation, migration, invasion, and survival of BC cells *in vitro*. **(A)** The qRT-PCR show the mRNA levels of TMPO-AS1 in five BC cell lines (BIU87, 5637, T24, EJ, and RT4). **(B)** The efficiencies of TMPO-AS1 knockdown or overexpression in RT4 and T24 cells were examined by qRT-PCR. Mock, the negative control; Si-TMPO-AS1, siRNA targeting TMPO-AS1; OE-TMPO-AS1, ectopic expression of TMPO-AS1. **(C,D)** The effect of TMPO-AS1 knockdown and overexpression on the cell proliferation was measured using methyl thiazolyl tetrazolium (MTT) and colony formation assays. **(E,F)** The effect of TMPO-AS1 knockdown or overexpression on the migration and invasion of RT4 and T24 cells was evaluated *via* wound healing and transwell assays. **(G)** Cell apoptosis was analyzed by flow cytometry in TMPO-AS1 knockout or overexpressing RT4 and T24 cells, stained with Annexin V-PE/7-AAD. Error bars represent the mean ± SD from three independent experiments. ^*^p < 0.05, ^**^p < 0.01, ^***^p < 0.001.

Since the expression of TMPO-AS1 was the highest in T24 and RT4 cells among five BC cell lines (BIU87, 5637, T24, EJ, and RT4) as per qRT-PCR (Figure 2A), these two cell lines were selected for the following experiments. To investigate the effects of TMPO-AS1 on the proliferation, migration, and apoptosis of BC cells, loss- and gain-of-function assays were performed. First, si-RNA targeting TMPO-AS1 or a TMPO-AS1-overexpression plasmid was transfected into T24 and RT4 cells. The transfection efficacy was examined using qRT-PCR (Figure 2B). Importantly, TMPO-AS1 knockdown significantly inhibited cell viability/proliferation (Figures 2C,D), migration (Figure 2E), and invasion (Figure 2F), whereas the overexpression of TMPO-AS1 resulted in the opposite effects. Additionally, cell apoptosis was induced in TMPO-AS1-silenced T24 and RT4 cells, whereas the overexpression of TMPO-AS1 inhibited cell apoptosis (Figure 2G). Overall, these findings suggest that TMPO-AS1 plays an oncogenic role in BC cells, promoting their proliferation, migration, invasion, and survival *in vitro*.

### E2F1 Activates the Transcription of TMPO-AS1 in BC Cells

**Figure 3:**
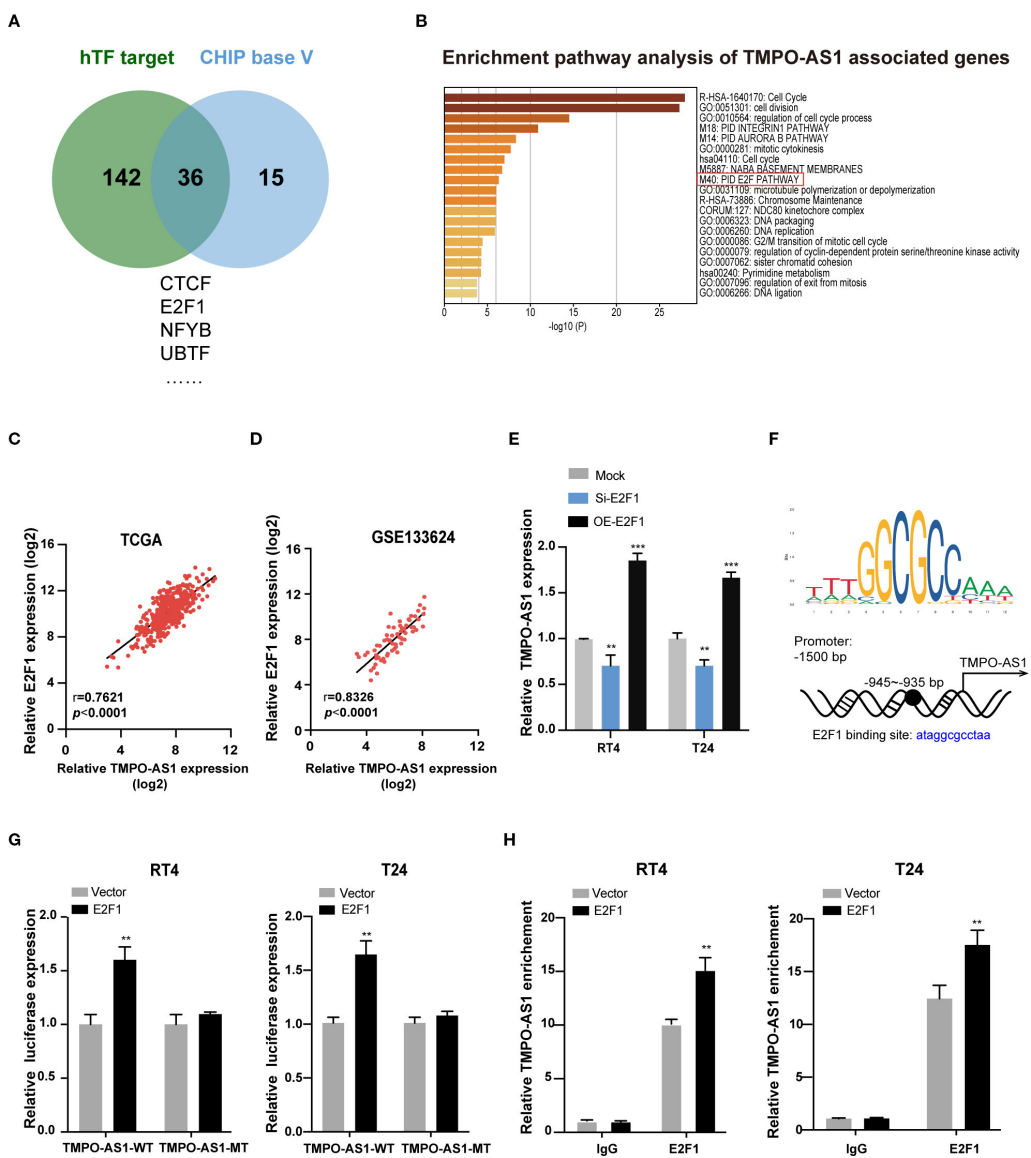
E2F1 binds to the promoter of TMPO-AS1 and activates its transcription. **(A)** The potential transcription factors of TMPO-AS1 were predicted using the ChIPBase v2.0 and hTFtarget. **(B)** The pathway enrichment analysis of genes co-expressed with TMPO-AS1 was obtained using Metascape. **(C,D)** The correlation between TMPO-AS1 and E2F transcription factor 1 (E2F1) mRNA expression in BC tissues was assessed using the Pearson's correlation analysis in datasets retrieved from TCGA and the GEO (GSE133624) databases. **(E)** The expression of TMPO-AS1 was evaluated using qRT-PCR in E2F1 silenced or overexpressing RT4 and T24 cells. Mock, the negative control; Si-E2F1, siRNA targeting E2F1; OE-E2F1, ectopic expression of E2F1. **(F)** The binding sites between E2F1 and the TMPO-AS1 promoter region were predicted using JASPAR. **(G)** Luciferase reporter assays were conducted to measure the TMPO-AS1 promoter luciferase activity in RT4 and T24 cells overexpressing E2F1. Vector, the negative control. **(H)** The ChIP assay demonstrates the binding between E2F1 and the TMPO-AS1 promoter. Error bars represent the mean ± SD from three independent experiments. ^**^p < 0.01, ^***^p < 0.001.

To figure out the underlying mechanism of TMPO-AS1-mediated carcinogenesis in BC, we investigated the upstream and downstream targets of TMPO-AS1. We screened out the potential TFs of TMPO-AS1 using hTFtarget and ChIPBase v2.0 (Figure 3A). Interestingly, we obtained 36 candidate TFs for TMPO-AS1; of note, E2F1 had the highest positive correlation with TMPO-AS1 among the 36 TFs (Supplementary Table 2). Importantly, a previous study reported that the overexpression of E2F3 induced the promoter activity of TMPO-AS1/LAP2, an antisense transcript (31). Additionally, we analyzed the genes co-expressed with TMPO-AS1 (correlation coefficient ≥0.6 and *p* < 0.01) in BC using Co-LncRNA (Supplementary Table 3) and used them in the context of pathway analysis *via* Metascape. Remarkably, we found that TMPO-AS1 is involved in the E2F pathway (Figure 3B). Thus, we focused on E2F1 in the following experiments, and we examine its transcriptional expression among five BC cell lines (Supplementary Figure 1B). Importantly, E2F1 was positively correlated with TMPO-AS1 in BC as evidenced by TCGA and GSE133624 datasets (Figures 3C,D). Furthermore, the expression of TMPO-AS1 was downregulated after E2F1 silencing and upregulated in E2F1-overexpressing BC cells (Figure 3E). Of note, the putative binding site between TMPO-AS1 and E2F1 was located at around −945 to −935 bp, upstream of the transcription start site, as predicted by JASPAR (http://jaspar.genereg.net), a collection of transcription factor binding site profiles (22) (Figure 3F). Importantly, this prediction was validated *via* luciferase reporter assays. The overexpression of E2F1 dramatically enhanced the luciferase activity of the wild-type TMPO-AS1 promoter but did not affect the transcriptional activity of the mutant TMPO-AS1 promoter (Figure 3G). Moreover, the results of ChIP assays demonstrated that E2F1 was remarkably enriched in the TMPO-AS1 promoter region relative to the observed in the context of control IgG (Figure 3H). Overall, these results indicate that E2F1 binds to the promoter of TMPO-AS1 and its transcription.

### TMPO-AS1 Regulates the Protein Levels of E2F1 *via* Protein Stabilization

**Figure 4:**
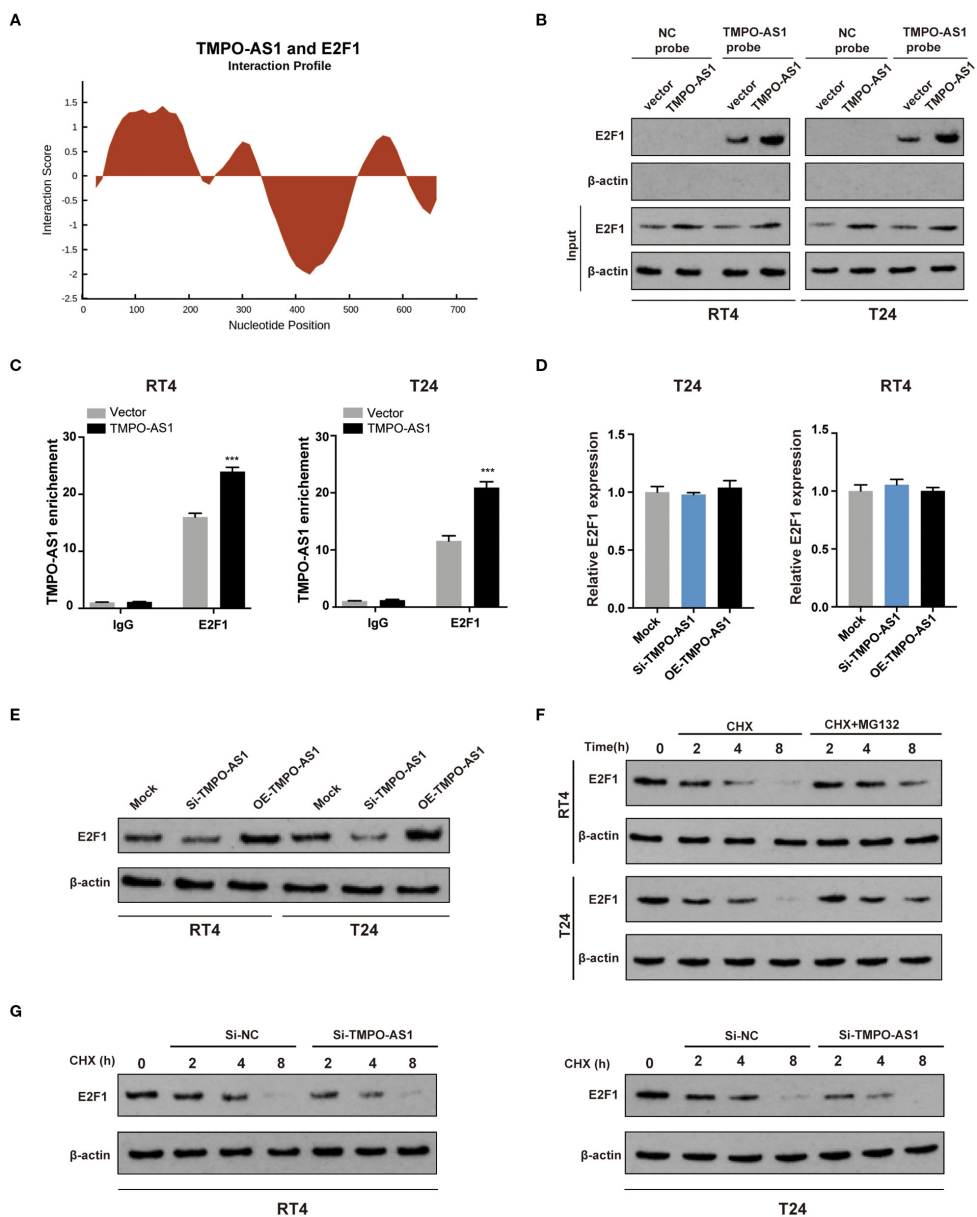
TMPO-AS1 upregulates the protein levels of E2F1 *via* the promotion of protein stability. **(A)** The interaction between TMPO-AS1 and E2F1 was predicted using catRAPID. **(B)** An RNA pull-down assay was performed to validate the potential interaction between TMPO-AS1 and E2F1 in RT4 and T24 cells overexpressing TMPO-AS1. **(C)** A radioimmunoprecipitation assay (RIPA) was performed to further confirm the interaction between E2F1 and TMPO-AS1 in TMPO-AS1 overexpressing RT4 and T24 cells; immunoglobulin G (IgG) was used as the negative control. Error bars represent the mean ± SD from three independent experiments. **(D)** *E2F1* mRNA expression was assessed using qRT-PCR in TMPO-AS1 knockout or overexpressing BC cells. **(E)** The E2F1 protein expression was evaluated *via* Western blotting in TMPO-AS1 knockout or overexpressing RT4 and T24 cells. **(F)** Western blotting show that the proteasome inhibitor MG132 protein increased the stability of E2F1 in cycloheximide-treated RT4 and T24 cells. **(G)** Western blotting showing the decreased E2F1 stability in TMPO-AS1 knockdown RT4 and T24 cells. CHX, cycloheximide. ^***^p < 0.001.

Given that lncRNAs have been reported to interact with E2F1 (32), we investigated whether TMPO-AS1 could interact with E2F1. As shown in Figure 4A, a possible interaction between TMPO-AS1 and E2F1 was predicted by catRAPID. Consequently, we conducted RNA pull-down assays to validate the prediction. The results showed that E2F1 was abundantly enriched in the context of the TMPO-AS1 probe compared with the oligo control, especially in TMPO-AS1 overexpressing RT4 and T24 cells (Figure 4B). Similarly, RIP results demonstrated that E2F1 remarkably immunoprecipitated TMPO-AS1, particularly after the TMPO-AS1 overexpression in RT4 and T24 cells (Figure 4C). The interaction between TMPO-AS1 and E2F1 prompted us to further investigate whether TMPO-AS1 influenced the expression of E2F1. Interestingly, the overexpression or knockdown of TMPO-AS1 had no significant impact on the E2F1 mRNA expression (Figure 4D), but it either increased or decreased the protein levels of E2F1 in RT4 and T24 cells (Figure 4E), respectively. These results suggest that the TMPO-AS1-mediated E2F1 regulation occurs at the posttranscriptional level. Of note, we found that the proteasome inhibitor MG132 markedly increased the stability of E2F1 in cycloheximide-treated RT4 and T24 cells (Figure 4F), implying that the turnover of E2F1 is dependent on the ubiquitin-proteasome system. Importantly, TMPO-AS1 silencing significantly shortened the half-life of E2F1 (Figure 4G). Altogether, our data demonstrate that TMPO-AS1 directly interacts with E2F1 and regulates its protein levels *via* protein stabilization.

### TMPO-AS1 Stabilizes E2F1 *via* OTUB1-Mediated Deubiquitination

**Figure 5:**
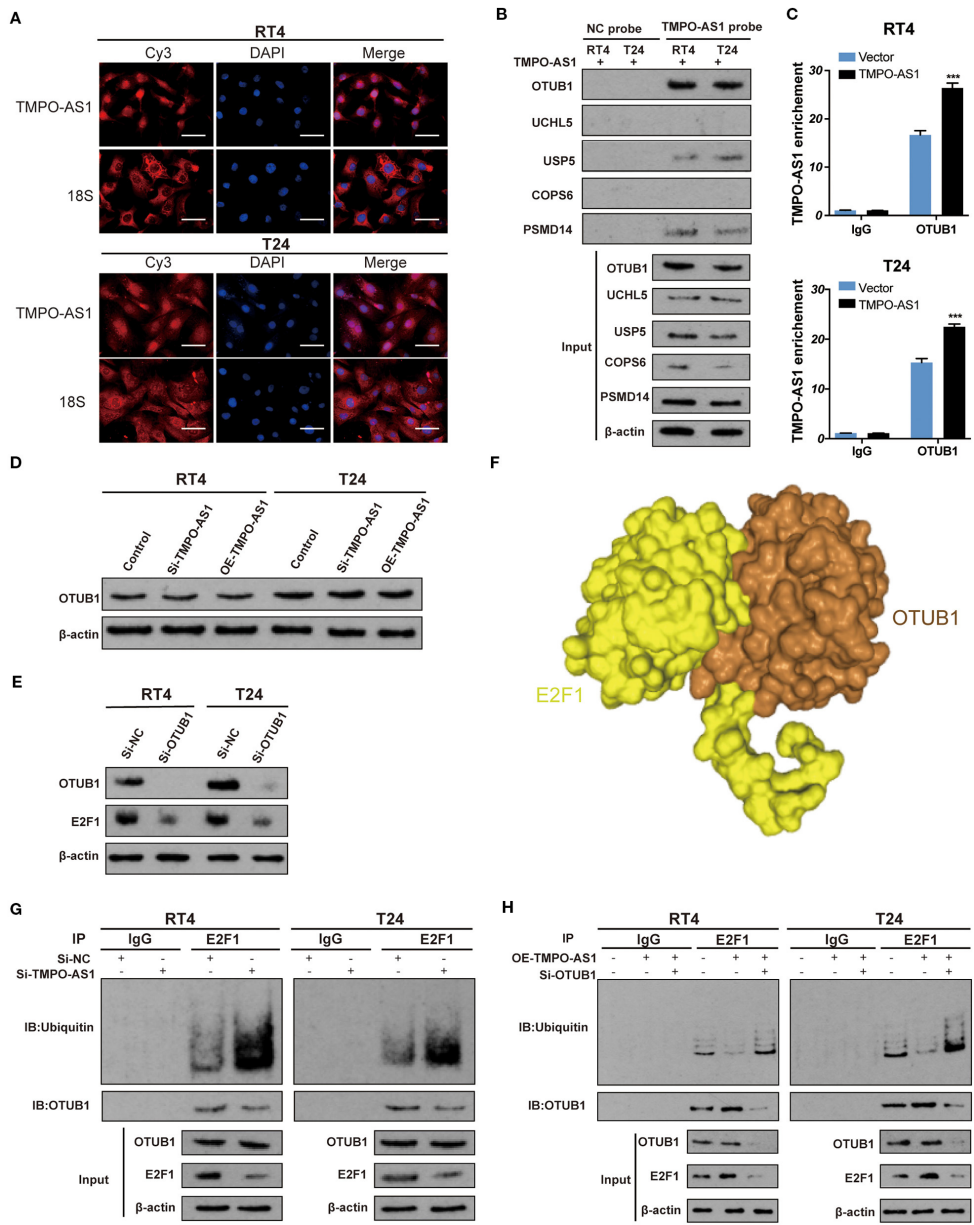
TMPO-AS1 stabilizes E2F1 *via* OTUB1-mediated deubiquitination. **(A)** The TMPO-AS1 cellular localization was evaluated using FISH. **(B)** RNA pull-down assays demonstrated that OTUB1 is the deubiquitinase that most likely binds to TMPO-AS among a group of deubiquitinases (OTUB1, UCHL5, USP5, COPS6, and PSMD14). **(C)** RIP assays were performed to validate the interaction between TMPO-AS1 and OTUB1, in the context of TMPO-AS1 overexpressing RT4 and T24 cells; IgG was used as the negative control. Error bars represent the mean ± SD from three independent experiments. **(D)** Western blotting images show that TMPO-AS1 silencing has no effect on the OTUB1 protein levels in RT4 and T24 cells. **(E)** Western blotting images showing that the knockdown of OTUB1 leads to the decrease in the E2F1 protein levels in RT4 and T24 cells. Si- OTUB1, siRNA targeting OTUB1. **(F)** E2F1 (yellow) and OTUB1 (brown) are very likely to bind to each other as per HDOCK predictions. **(G)** Co-IP assays were performed in control or si-TMPO-AS1-treated RT4 and T24 cells using an anti-E2F1 antibody, followed by Western blotting to analyze the ubiquitin levels of E2F1. **(H)** RT4 and T24 cells transfected with the empty vector, and the construct for the overexpression of TMPO-AS1, alone, or together with si-OTUB1 were subjected to immunoprecipitation with an anti-E2F1 antibody, followed by Western blotting to analyze the ubiquitin levels of E2F1. ^***^p < 0.001.

Later, we aimed to understand the underlying mechanism of TMPO-AS1-mediated E2F1 stabilization. First, using FISH, we found that TMPO-AS1 is predominantly distributed in the cytoplasm in both T24 and RT4 cells (Figure 5A), indicating that TMPO-AS1 might be involved in translational regulation. Of note, it was predicted that E2F1 has four potential ubiquitination sites, as per UbPred (Supplementary Figure 2A). Therefore, we hypothesized that TMPO-AS1 may associate with a specific ubiquitinase/deubiquitinase to regulate E2F1 ubiquitination. Since we have previously studied a group of deubiquitinases (OTUB1, UCHL5, USP5, COPS6, and PSMD14) in BC (unpublished data), we evaluated their binding potential to TMPO-AS1 using PRIdictor. The results highlighted OTUB1 as the deubiquitinase that most likely associates with TMPO-AS1 (Supplementary Figure 2B), and the transcriptional expression of OTUB1 was evaluated by qRT-PCR (Supplementary Figure 1C). To validate this prediction, we conducted RNA pull-down assays and found that TMPO-AS1 precipitated more OTUB1 than the other deubiquitinases (Figure 5B). Similarly, RIP assays showed that TMPO-AS1 was obviously immunoprecipitated by the anti-OTUB1 antibody (vs. IgG; Figure 5C). Furthermore, catRAPID showed that a region of TMPO-AS1 (located at 76–127 nt) exhibits a high potential of interaction with some of the OTUB1 amino acid residues (51–152; Supplementary Figure 2C), further supporting the association between TMPO-AS1 and OTUB1. Of note, despite the direct association between TMPO-AS1 and OTUB1, TMPO-AS1 failed to alter the OTUB1 protein expression (Figure 5D). Importantly, the OTUB1 knockdown led to a decrease in the E2F1 protein levels in RT4 and T24 cells (Figure 5E), suggesting an interaction between these two proteins. Such interaction was further supported by the prediction by HDOCK; OTUB1 is very likely to bind to E2F1 (Figure 5F). Importantly, the results of co-immunoprecipitation (Co-IP) assays showed that silencing TMPO-AS1 not only increased E2F1 ubiquitination but also mitigated the interaction between OTUB1 and E2F1 (Figure 5G); importantly, this phenotype was rescued after the TMPO-AS1 overexpression. Furthermore, the OTUB1 knockdown significantly reversed the decreased ubiquitination of E2F1 induced by the overexpression of TMPO-AS1 in RT4 and T24 cells (Figure 5H). Taken together, these results indicate that TMPO-AS1 upregulates E2F1 protein levels *via* OTUB1-mediated deubiquitination and the consequent protein stabilization.

### E2F1 Promotes the Proliferation, Migration, and Invasion and Inhibits the Apoptosis of BC Cells *in vitro*

**Figure 6:**
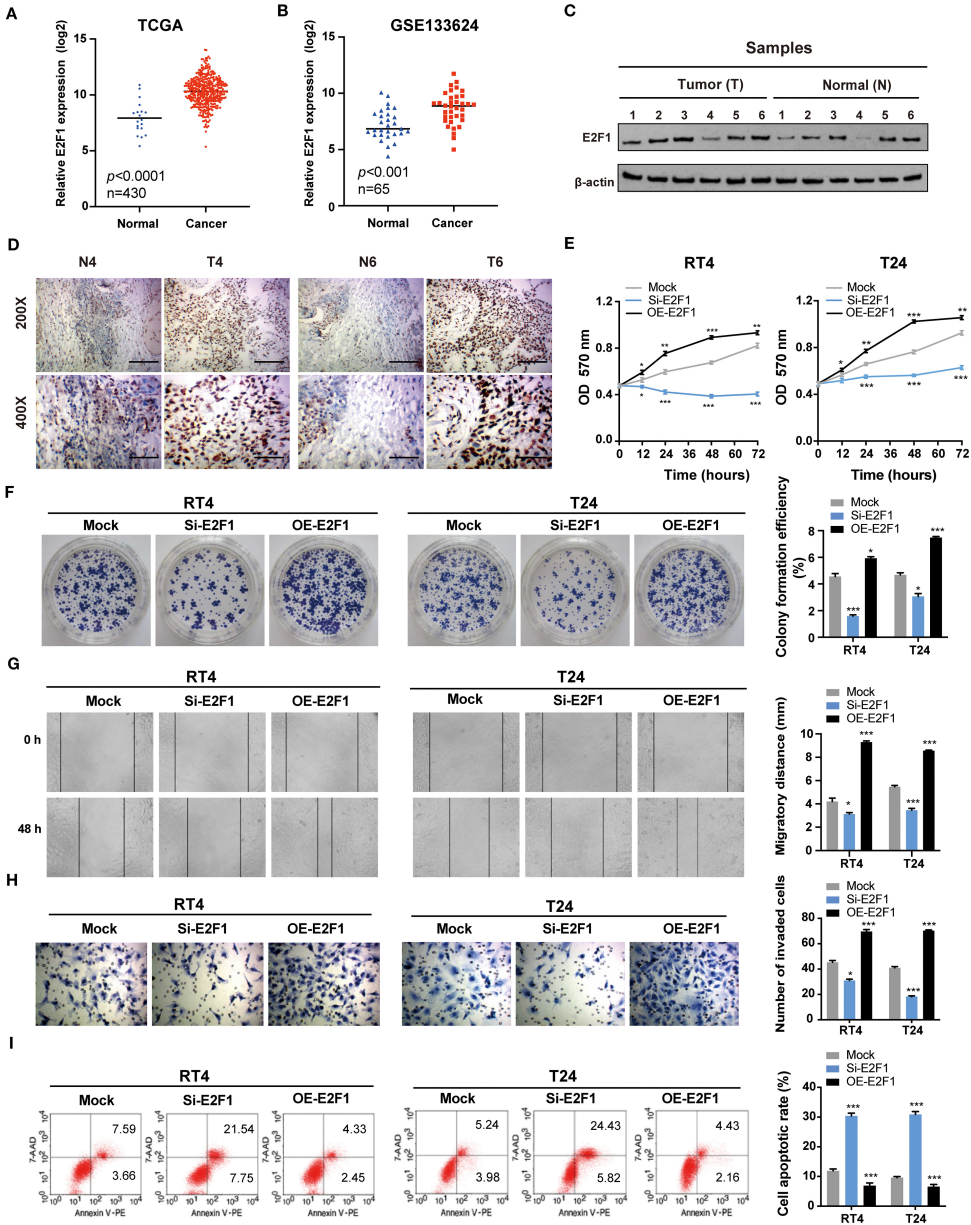
E2F1 promotes the proliferation, migration, and invasion and inhibits the apoptosis of BC cells *in vitro*. **(A,B)** The expression of *E2F1* is upregulated in BC tissues compared with that in normal tissues based on data retrieved from TCGA and the GEO (GSE133624) databases. **(C)** The protein expression of E2F1 was assessed *via* Western blotting in six paired BC and adjacent normal tissue samples. **(D)** The protein expression of E2F1 was also assessed using immunohistochemistry in six paired BC and adjacent normal tissue samples. **(E)** The MTT assay was performed to determine the viability/proliferation of E2F1 knockdown and overexpressing RT4 and T24 cells. **(F)** The effects of E2F1 knockdown and overexpression on BC cell proliferation were assessed *via* colony formation assays. **(G,H)** The migratory and invasive capacities of E2F1 knockdown and overexpressing RT4 and T24 cells were evaluated *via* wound healing and transwell assays. **(I)** Cell apoptosis was analyzed by flow cytometry. Error bars represent the mean ± SD from three independent experiments. ^*^p < 0.05, ^**^p < 0.01, ^***^p < 0.001.

E2F1 is associated with cell proliferation, apoptosis, metastasis, and invasiveness (33). Hence, we speculated that E2F1 would promote BC tumorigenesis and development. According to the data retrieved from TCGA and the GEO (GSE133624) databases, the expression of E2F1 was remarkably higher in BC tissues than that in normal tissues (Figures 6A,B). Furthermore, Western blotting and immunohistochemistry (IHC) analyses showed that the expression of E2F1 protein was upregulated in six paired BC tissues compared with the adjacent normal tissues (Figures 6C,D). As anticipated, further functional experiments demonstrated that the E2F1 knockdown significantly inhibited cell proliferation (Figures 6E,F), migration (Figure 6G), and invasion (Figure 6H), and induced apoptosis (Figure 6I), whereas the overexpression of E2F1 led to the opposite effects. Altogether, these results reveal that E2F1 promotes malignant phenotypes in BC cells.

### TMPO-AS1 Regulates Malignant Phenotypes in BC Cells *via* E2F1 *in vitro*

**Figure 7:**
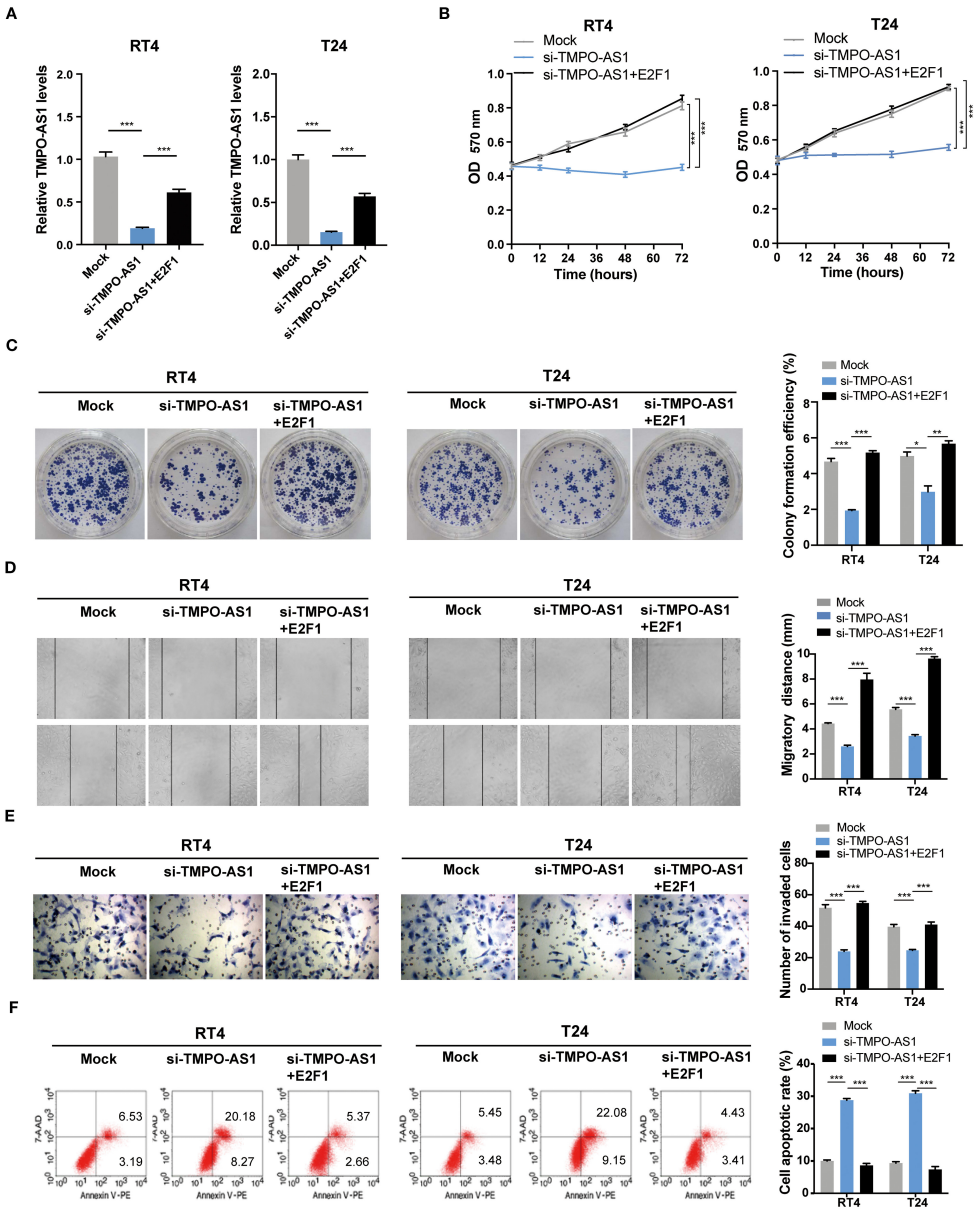
TMPO-AS1 promotes BC malignant phenotypes *via* E2F1 *in vitro*. **(A)** The TMPO-AS1 expression was evaluated using qRT-PCR in RT4 and T24 cells transfected with an empty vector, sh-TMPO-AS1, or co-transfected with sh-TMPO-AS1 and E2F1-ectopic expression vector. **(B,C)** MTT and colony formation assays were performed to assess the proliferative ability of transfected cells. **(D,E)** The cell migration and invasion were estimated using wound healing and transwell assays. **(F)** The apoptosis in transfected BC cells stained with Annexin V-PE/7-AAD was evaluated *via* flow cytometry analysis. Error bars represent the mean ± SD from three independent experiments. ^*^p < 0.05, ^**^p < 0.01, ^***^p < 0.001.

Since the overexpression of either E2F1 or TMPO-AS1 enhanced the proliferation, migration, and invasion of BC cells, and TMPO-AS1 upregulated the protein levels of E2F1, we hypothesized that TMPO-AS1 would promote BC progression *via* E2F1. To prove this theory, we restored the expression of E2F1 in TMPO-AS1-silenced BC cells (Figure 7A) and performed MTT, colony formation, wound healing, and transwell assays. As expected, the restoration of E2F1 in BC cells significantly reversed the inhibition of cell proliferation (Figures 7B,C), migration (Figure 7D), and invasion (Figure 7E) induced by TMPO-AS1 silencing. Furthermore, the TMPO-AS1 silencing-mediated promotion of apoptosis could be abrogated by the E2F1 overexpression (Figure 7F). Therefore, our data demonstrate that TMPO-AS1 promotes cell proliferation, migration, invasion, and survival of BC *via* E2F1 *in vitro*.

### TMPO-AS1 Regulates BC Growth *via* E2F1 *in vivo*

**Figure 8:**
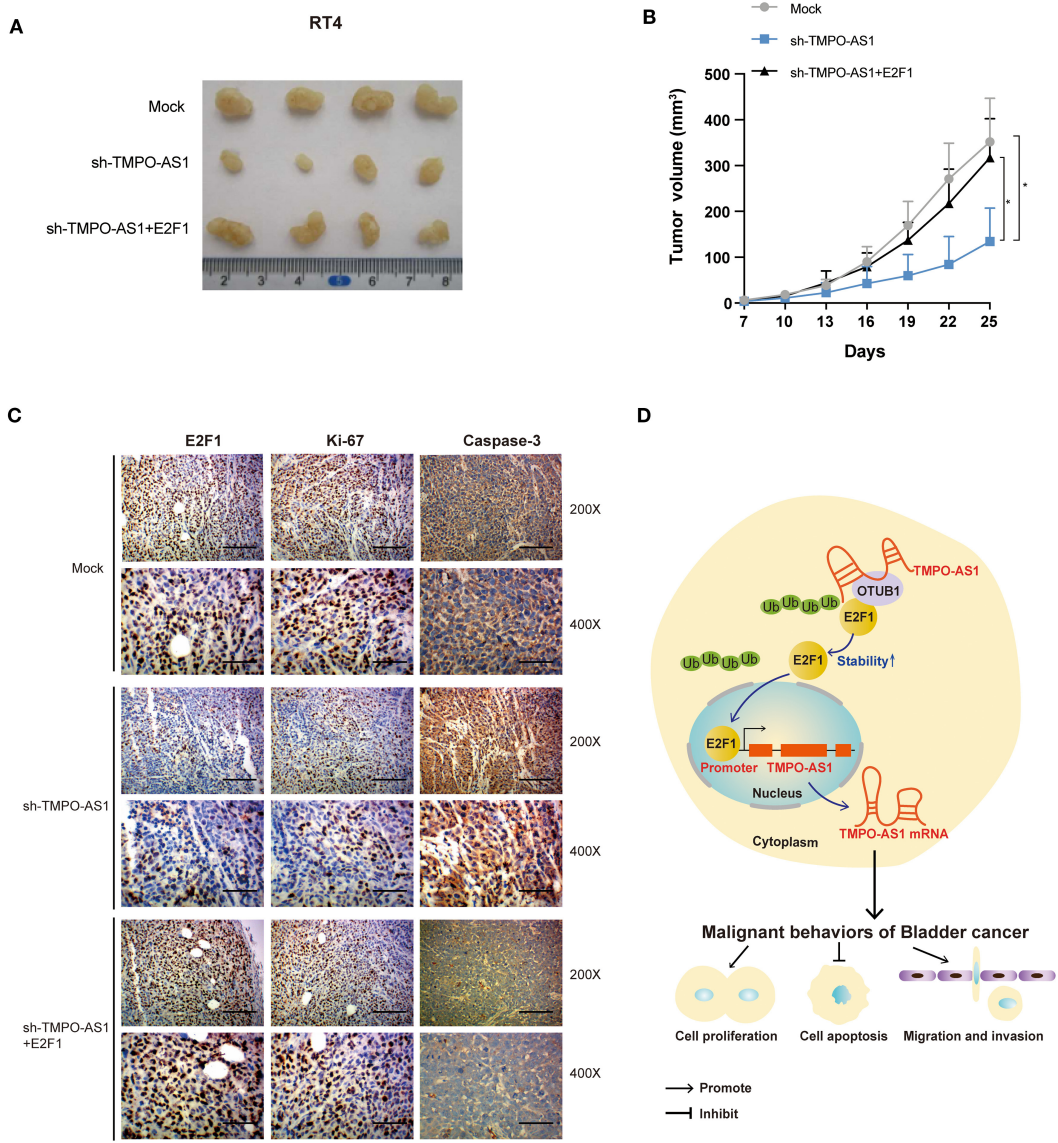
TMPO-AS1 regulates BC growth *in vivo via* E2F1. **(A)** Representative images of the tumor xenografts 25 days after the subcutaneous injection of RT4 cells transfected with empty vector, sh-TMPO-AS1, and sh-TMPO-AS1 along with an E2F1-overexpression construct into the flanks of nude mice. **(B)** The tumor volumes were measured every 3 days in the three groups. Three independent experiments were performed. **(C)** The expression of E2F1, Ki-67, and caspase-3 in xenografts was evaluated using immunohistochemistry. Representative images are shown. **(D)** A schematic diagram illustrating the role and mechanism of TMPO-AS1 in BC tumorigenesis and progression is illustrated. ^*^p < 0.05.

Stably transfected RT4 cells were subcutaneously inoculated into nude mice to explore whether TMPO-AS1 would promote BC growth *via* E2F1 *in vivo*; every 5 days, the tumor volumes were measured. Results showed that the knockout of TMPO-AS1 significantly suppressed tumor growth compared to the control group, whereas the overexpression of E2F1 abolished the inhibitory effect on tumor growth induced by the knockout of TMPO-AS1 (Figures 8A,B). Furthermore, the IHC staining showed that the depletion of TMPO-AS led to a substantial decrease of the protein levels of Ki-67 and E2F1 and to a notable increase in the expression of caspase-3. Of note, this phenotype was reversed by the overexpression of E2F1 (Figure 8C). Collectively, the above findings demonstrate that TMPO-AS1 regulates the BC-associated tumor growth through E2F1 *in vivo*.

## Discussion

Bladder cancer is the most common urinary system malignancy, with a heavy burden worldwide. Therefore, it is imperative to elucidate the mechanisms of carcinogenesis in BC. An increasing number of studies have demonstrated that lncRNAs play a vital role in tumor initiation, development, and progression in the context of numerous cancers, including BC (34). In our study, we investigated the biological roles of the lncRNA TMPO-AS1 in BC. Functional experiments demonstrated that TMPO-AS1 could promote the proliferation, migration, and invasion of BC cells and could inhibit apoptosis in BC cells *via* the stabilization of E2F1.

Here, we found that TMPO-AS1 is highly expressed in BC and correlates with poor prognoses. Importantly, we performed *in vitro* experiments and proved that TMPO-AS1 functions as an oncogene, consistent with the reported in previous studies (35). In fact, thousands of abnormally expressed lncRNAs have been reported in BC (36). However, little is known about their upstream regulation and downstream targets. In the present study, we showed that TMPO-AS1 positively correlates with the expression of E2F1; moreover, we show that E2F1 activates the transcription of TMPO-AS1. Therefore, our data, showing that TMPO-AS1 is upregulated in BC due to E2F1, contribute to a better understanding of the upstream regulatory mechanisms in the context of lncRNAs.

LncRNAs may be oncogenes or tumor suppressors *via* the regulation of gene expression (e.g., epigenetic regulation, transcriptional activation or repression, or posttranscriptional modulation) or even *via* protein modification (6, 37). Our data demonstrated that TMPO-AS1 directly binds to E2F1, increasing its stability. The knockdown of TMPO-AS1 resulted in higher E2F1 ubiquitination levels. Interestingly, we demonstrated that OTUB1, a deubiquitinase, is responsible for the TMPO-AS1-mediated E2F1 stabilization. In line with our results, a previous study revealed that POH1, a deubiquitinase, binds to and deubiquitinates E2F1, contributing to its stabilization (38).

Most studies suggest that TMPO-AS1 contributes to tumorigenesis through TMPO-AS1–miRNA–mRNA axes (39), whereas others propose that TMPO-AS1 forms RNA–RNA complexes to regulate the gene expression (40). In fact, very recently, a study has shown that TMPO-AS1 promotes BC cell growth *via* the TMPO-AS1/miR-98-5p/EBF1 positive feedback loop (41). However, this study only elucidated the function of TMPO-AS1 as a miRNA “sponge” for *EBF1* mRNA *in vitro*; the roles of TMPO-AS1 in BC, *in vivo*, are still unknown. Therefore, our study highlights, for the first time, that TMPO-AS1, to act as a “sponge” for mRNAs, interacts directly with proteins and, thereby, influences protein–protein interactions, revealing a novel regulatory mechanism of TMPO-AS1.

Additionally, in the current study, we found that the overexpression of either E2F1 or TMPO-AS1 boosts the proliferation, migration, and invasion and inhibits apoptosis of BC cells. Moreover, we verified that TMPO-AS1 promotes BC growth and progression in an E2F1-dependent manner. In fact, numerous studies have shown that lncRNAs can target E2F1 in cancers, such as lung carcinoma (42), breast cancer (32), and BC (43). Moreover, it is well acknowledged that E2F1 can promote tumor carcinogenesis, development, and progression *via* the facilitation of cell proliferation (44) and migration (45, 46). Of note, upregulated E2F1 is a strong predictor of the BC progression (33). Together with these previous studies, our data support the notion that that the positive feedback loops are extremely important for the regulation of carcinogenesis and cancer progression (43, 47). Particularly, our data highlight a mutual regulatory pattern between lncRNAs and TFs, which may lead to increased oncogenic activity in cancer development.

However, our study is not without limitations. Due to the low number of available clinical BC samples, we validated the clinical significance of TMPO-AS1 using the online software. In addition, the specific mechanistic sequence behind the TMPO-AS1-induced OTUB1-mediated E2F1 deubiquitination, as well as the structure of the possible complex formed, remains unknown. We plan to investigate the abovementioned detail in a follow-up study.

In summary, our study uncovers the upstream and downstream partners of TMPO-AS1 in the context of BC, proposing a new positive feedback loop that contributes to BC tumorigenesis, involving TMPO-AS1 and E2F1 (Figure 8D). Therefore, the TMPO-AS1/E2F1 loop should be considered in the development of new therapeutic approaches for BC.

## Data Availability Statement

The original contributions presented in the study are included in the article/Supplementary Material, further inquiries can be directed to the corresponding author/s.

## Ethics Statement

The studies involving human participants were reviewed and approved by ethics committee of the Third Xiangya Hospital, Central South University. The patients/participants provided their written informed consent to participate in this study. The animal study was reviewed and approved by Animal Care and Use Committee of the Central South University. Written informed consent was obtained from the individual(s) for the publication of any potentially identifiable images or data included in this article.

## Author Contributions

KC and YYZ designed this study. JL and MX collected the clinical samples. JL and YYZ contributed to the experiments implementation. YC, DH, and LX analyzed the data. KC, YYZ, and YXZ drafted the manuscript. LG, ZW, and LD edited the manuscript. All authors read and approved the final manuscript.

## Conflict of Interest

The authors declare that the research was conducted in the absence of any commercial or financial relationships that could be construed as a potential conflict of interest.

## Supplementary Material

Supplementary Figure 1 The expression of TMPO-AS1, E2F1, and OTUB1 in bladder cancer (BC). **(A)** The expression of TMPO-AS1 in BC samples in the context of different clinical stages. **(B)** The mRNA levels of E2F1 in five BC cell lines (BIU87, 5637, T24, EJ, and RT4). **(C)** Quantitative real-time PCR (qRT-PCR) showing the mRNA levels of OTUB1 in five BC cell lines (BIU87, 5637, T24, EJ, and RT4).

Supplementary Figure 2 E2F1 ubiquitination sites and the interactions between TMPO-AS1 and five deubiquitinases. **(A)** The potential ubiquitination sites in E2F1. **(B)** The interaction between TMPO-AS1 and five deubiquitinases (UCHL5, USP5, COPS6, PSMD14, and OTUB1) predicted by PRIdictor. **(C)** CatRAPID was used to predict the interaction pattern between TMPO-AS1 and OTUB1.

Supplementary Table 1 List of antibodies used in this study.

Supplementary Table 2 The 36 overlapping transcription factors (TFs) revealed by the hTFtarget and ChIPBase v2.0 and the correlation analysis between their expression and TMPO-AS1.

Supplementary Table 3 Genes co-expressed with TMPO-AS1 as per Co-lncRNA.
