# Supplementary figures and images for: The Long Non-coding RNA TMPO-AS1 Promotes Bladder Cancer Growth and Progression via OTUB1-Induced E2F1 Deubiquitination
Source: Front Oncol. 2021 Mar 18;11:643163. doi: 10.3389/fonc.2021.643163 (PMC8013732; doi:10.3389/fonc.2021.643163)

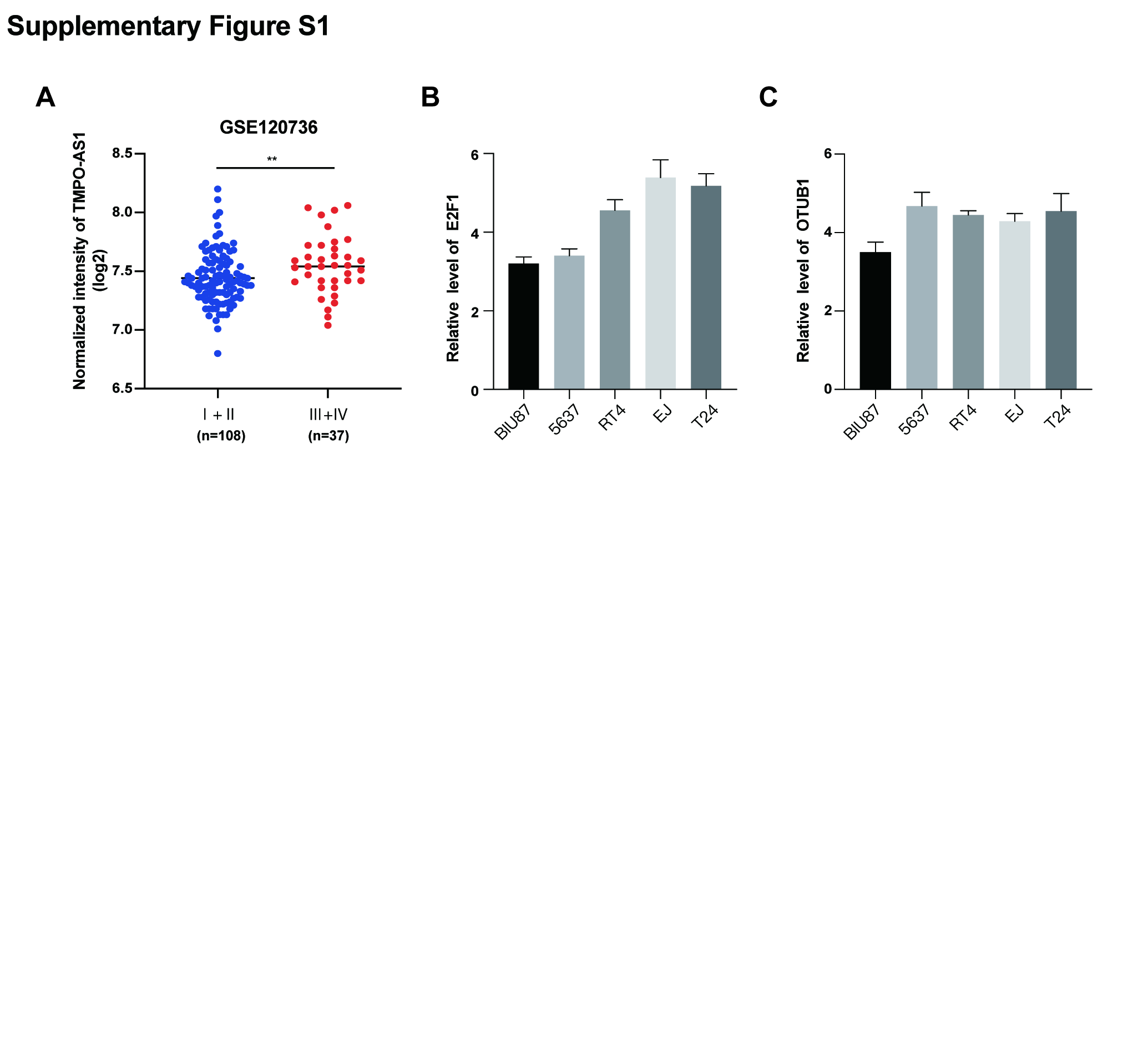

Supplement: Supplementary Figure 1 — The expression of TMPO-AS1, E2F1, and OTUB1 in bladder cancer (BC). (A) The expression of TMPO-AS1 in BC samples in the context of different clinical stages. (B) The mRNA levels of E2F1 in five BC cell lines (BIU87, 5637, T24, EJ, and RT4). (C) Quantitative real-time PCR (qRT-PCR) showing the mRNA levels of OTUB1 in five BC cell lines (BIU87, 5637, T24, EJ, and RT4). [file Image_1.TIF]

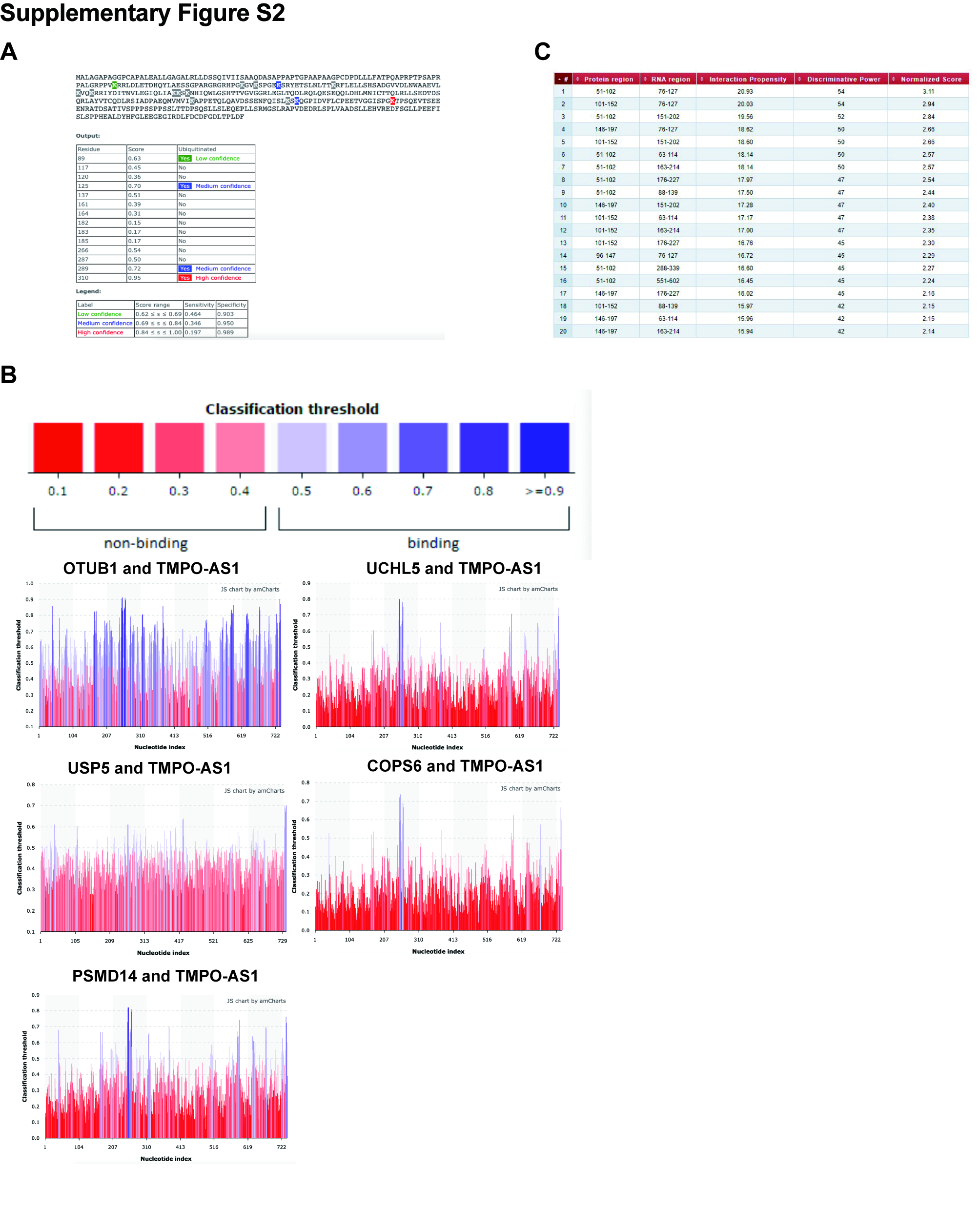

Supplement: Supplementary Figure 2 — E2F1 ubiquitination sites and the interactions between TMPO-AS1 and five deubiquitinases. (A) The potential ubiquitination sites in E2F1. (B) The interaction between TMPO-AS1 and five deubiquitinases (UCHL5, USP5, COPS6, PSMD14, and OTUB1) predicted by PRIdictor. (C) CatRAPID was used to predict the interaction pattern between TMPO-AS1 and OTUB1. [file Image_2.TIF]
