# Supplementary material for: The Long Non-coding RNA TMPO-AS1 Promotes Bladder Cancer Growth and Progression via OTUB1-Induced E2F1 Deubiquitination
Source: Front Oncol. 2021 Mar 18;11:643163. doi: 10.3389/fonc.2021.643163 (PMC8013732; doi:10.3389/fonc.2021.643163)
Supplement: Supplementary Table 1 — List of antibodies used in this study. [file Data_Sheet_1.zip › Supplementary Table 2.DOCX]

**Table S2. Overlapping TFs in hTFtarget and ChIPBase v2.0 databases**

| **TFs** | **Correlation with TMPO-AS1** | |
| --- | --- | --- |
| CTCF | | 0.44 |
| E2F1 | | 0.64 |
| NFYB | | 0.51 |
| UBTF | | 0.43 |
| YY1 | | 0.42 |
| E2F6 | | 0.37 |
| SP1 | | 0.33 |
| GABPA | | 0.31 |
| TCF12 | | 0.31 |
| RCOR1 | | 0.3 |
| ARNT | | 0.28 |
| CREB1 | | 0.25 |
| E2F4 | | 0.19 |
| NFYA | | 0.19 |
| EBF1 | | 0.18 |
| TFAP2C | | 0.17 |
| SRF | | 0.14 |
| PAX5 | | 0.14 |
| FOXA1 | | 0.13 |
| RELA | | 0.12 |
| TP53 | | 0.11 |
| EGR1 | | 0.1 |
| STAT3 | | 0.1 |
| ELF1 | | 0.037 |
| VDR | | 0.019 |
| NANOG | | -0.008 |
| ETS1 | | -0.02 |
| IRF3 | | -0.024 |
| MYC | | -0.046 |
| ZBTB7A | | -0.058 |
| ERG | | -0.06 |
| SPI1 | | -0.063 |
| FOS | | -0.071 |
| ZEB1 | | -0.075 |
| CEBPB | | -0.078 |
| GATA1 | | -0.1 |
